# Dipstick Test for Rapid Diagnosis of *Shigella dysenteriae* 1 in Bacterial Cultures and Its Potential Use on Stool Samples

**DOI:** 10.1371/journal.pone.0024830

**Published:** 2011-10-03

**Authors:** Neelam Taneja, Faridabano Nato, Sylvie Dartevelle, Jean Marie Sire, Benoit Garin, Lan Nguyen Thi Phuong, Tai The Diep, Jean Christophe Shako, François Bimet, Ingrid Filliol, Jean-Jacques Muyembe, Marie Noëlle Ungeheuer, Catherine Ottone, Philippe Sansonetti, Yves Germani

**Affiliations:** 1 Department of Medical Microbiology, Postgraduate Institute of Medical Education and Research, Chandigarh, India; 2 Plate-Forme 5 - Production de Protéines recombinantes et d'Anticorps, Institut Pasteur, Paris, France; 3 Laboratoire de Biologie Médicale, Institut Pasteur de Dakar, Dakar, Sénégal; 4 Department of Immunology and Microbiology, Pasteur Institute of Ho Chi Minh City, Ho Chi Minh City, Vietnam; 5 Antenne Médicale de Bunia, Bunia, République Démocratique du Congo; 6 Centre de Ressources Biologiques, Institut Pasteur, Paris, France; 7 Centre National de Référence des Escherichia coli et Shigella, Unité de Recherche et d'Expertise des Bactéries Pathogènes Entériques, Institut Pasteur, Paris, France; 8 Institut National de Recherche Biomédicale, Kinshasa, République Démocratique du Congo; 9 Plate-forme Investigation Clinique et Accès aux Ressources Biologiques, Institut Pasteur, Paris, France; 10 Unité de Pathogénie Microbienne Moléculaire, Institut Pasteur, Paris, France; 11 BioSpeedia Société par Actions Simplifée, Orsay, France; University of Delhi, India

## Abstract

**Background:**

We describe a test for rapid detection of *S. dysenteriae* 1 in bacterial cultures and in stools, at the bedside of patients.

**Methodology/Principal Findings:**

The test is based on the detection of *S. dysenteriae* 1 lipopolysaccharide (LPS) using serotype 1-specific monoclonal antibodies coupled to gold particles and displayed on a one-step immunochromatographic dipstick. A concentration as low as 15 ng/ml of LPS was detected in distilled water and in reconstituted stools in 10 minutes. In distilled water and in reconstituted stools, an unequivocal positive reaction was obtained with 1.6×10^6^ CFU/ml and 4.9×10^6^ CFU/ml of *S. dysenteriae* 1, respectively. Optimal conditions to read the test have been determined to limit the risk of ambiguous results due to appearance of a faint yellow test band in some negative samples. The specificity was 100% when tested with a battery of *Shigella* and unrelated strains in culture. When tested on 328 clinical samples in India, Vietnam, Senegal and France by laboratory technicians and in Democratic Republic of Congo by a field technician, the specificity (312/316) was 98.7% (95% CI:96.6–99.6%) and the sensitivity (11/12) was 91.7% (95% CI:59.8–99.6%). Stool cultures and the immunochromatographic test showed concordant results in 98.4 % of cases (323/328) in comparative studies. Positive and negative predictive values were 73.3% (95% CI:44.8–91.1%) and 99.7% (95% CI:98–100%).

**Conclusion:**

The initial findings presented here for a simple dipstick-based test to diagnose *S. dysenteriae* 1 demonstrates its promising potential to become a powerful tool for case management and epidemiological surveys.

## Introduction

*Shigella* is one of the most common bacterial agents of acute diarrhoea. It has been estimated that 165 million cases of shigellosis occur annually worldwide, resulting in 1.1 million deaths, mainly in the Third World [1], [2]–[5]. Among the numerous *Shigella* serotypes, *S. dysenteriae* type 1 was the first described and stands out for causing deadly epidemics in the most impoverished areas, particularly in situation of natural disaster or war (i.e. refugees camps, forced human displacement). A severity of symptoms, high attack rate, high case-fatality rate in all age groups [6]–[12] but especially in children under 5 years [7], and various complications [13] are hallmark characteristics of infection with the Shiga bacillus. This bacterium was responsible for large dysentery epidemics in Guatemala and other parts of Zaire [14], Central America [15], Bangladesh [16], Kenya [17], and recently West Africa [18] and India [19]. In the last decade, epidemic *S. dysenteriae* 1 strains resistant to ampicillin, chloramphenicol, nalidixic acid, tetracycline, trimethoprim-sulfamethoxazole, and only moderately susceptible to ciprofloxacin have been isolated with increasing frequency in Africa and Asia [10], [19], [20], [21], while strains resistant to ciprofloxacin have recently been isolated in India and Bangladesh, thus reducing the availability of effective oral therapy [16], [17].

Early diagnosis of acute enteric infections is currently a significant clinical problem especially in areas of conflict or mass displacement of susceptible persons, remains challenging [19], [20]. Rapid test methods, in which the response is achieved relatively quickly, are gaining in importance when timely antiepidemic measures (quarantine, disinfection, examination of contacts) and proper etiotropic therapy are needed [18], [19], [20]. Most rapid test methods currently practiced have been devised to search for antibodies to infectious agents in blood or in other biological fluids of patients, and only some have been tailored for direct identification of the pathogen. Obviously, these latter are more favored from an epidemiologic standpoint. The rapid and sensitive diagnosis of *S. dysenteriae* 1 is essential to be able to immediately treat the patients, to provide chemoprophylaxis to the subjects in contact with the pathogen, and to implement control measures to stop microbial transmission. In order to achieve this, improved diagnostic tools are needed to complete the currently used classical microbiological methods. Such tests should be robust, quick, reliable (sensitive and specific), efficient on fecal samples and easy to use at the patient's bedside or in the field.

Immunoassays are extremely simple to perform and have become more common at resource-limited sites, particularly in the form of lateral flow immunochromatographic devices [22]. Immunochromatographic techniques using dipsticks are based on the recognition of pathogen-specific antigens by monoclonal antibodies (mAbs). Such dipsticks have already been successfully developed at Institut Pasteur for cholera [23], meningitidis [24], plague [25] and *S. flexneri* 2a [26]. In this study, we investigated the potential of the dipstick technology to detect *S. dysenteriae* 1 in bacterial cultures and in stools. The dipstick is based on the detection of lipopolysaccharide (LPS), the major bacterial surface antigen. Indeed, *Shigella* serotypes are defined by the structure of the oligosaccharide repeating unit (RU) that forms the O-antigen (O-Ag), the polysaccharide moiety of LPS [27]. The O antigen of *S. dysenteriae* type 1 is essential for virulence and there is indirect evidence that antibodies against the O antigen are protective [28], [29]. The antigen consists of tetrasaccharide repeating units of the following structure: R3)-α-L-Rhap-(1R3)- α-L-Rhap-(1R2)-α-DGalp-(1R3)-α-D-GlcpNAc-(1R [30].

In this preliminary study, we demonstrate that a dipstick based on mAbs recognition of serotype 1-specific determinants carried by the LPS O-Ag, is a rapid, robust and reliable test to identify *S. dysenteriae* 1 in bacterial cultures when used by laboratory technicians. It has also been evaluated on stools in different settings by laboratory and field technicians (dispensaries) with encouraging results.

## Materials and Methods

### 1. Ethics Statement

In India, Vietnam, Senegal and France, written informed consent was obtained from all participants involved in the study. This study was approved by the Institute Ethics Committee of Postgraduate Institute of Medical education and Research in Chandigarh (institutional review board included Girish Varshney, Jatinder Mohan, Kusum Joshi, Sudesh Prabhakar, Rajesh Kumar, Jai Dev Wig, Niranjan Khandelwal, Sanjay Jain, Sunil Arora, Nirmal Kumar Ganguly, Prem Kumar Palli, Arunaloke Chakrabarti), by the Ethics Committee of Senegal (national review board included Moustapha Dieng, Djiby Faye, Cheikh Mbacké Lô, Aïssatou Touré, Charles Becker, Alassane Wade, Ismaila Goudiaby, Aldiouma Diallo, Malick Cissé, Mamadou Lamine Sow, Ami Collé Gueye, Birame Dramé, Samba Cor Sarr, Mamadou Lamine Badji, Pape Touré, Léon Diouf, Anta Tall Dia) and by the Scientific and Ethical Committee of Pasteur Institute in Ho Chi Minh City (institutional review board included Nguyen Van Tam, Nguyen Kim Dung, Nguyen Thi Nguyet Thu, Cao Minh Thang, Ho Thi Thien Ngan). In France, feces from healthy donors were supplied by the Platform ICAReB through the cohort project "Diagmicoll". This protocol, designed to set up new diagnostic methods for infectious diseases, has been approved by the French Ethical Committee (CPP Ile-de-France I including Elisabeth Frija-Orvoën, Nadine Forest, Marc Delpech, Christophe Bardin, Catherine Grillot-Courvalin, François Dauchy, Cécile Koronkiewcz, Jean-Michel Zucker, Lydia Morin, Catherine Labrusse-Riou, Antoine Fourment, Marie-Annick Cornu-Thenard, Pierre Frantz) and declared to the Research Ministry under the code n°DC 2008-68. In the DRC all specimens were collected as part of the routine clinical management of patients according to the national guidelines; Institut National de Recherche Biomédicale exempted this part of the study from review because the circumstances (epidemics); verbal informed consent was obtained from each patient; the process was documented on laboratory booklet and the Ethics Committee of the DRC (institutional review board included Ndelo Di Phanzu, Nguma Monganza, Kashala Tumba Diog, Okitolonda Wemakoy, Munyanga Mukungo, Kiyombo Mbela, Tshefu Kitoto, Kayembe Kalambayi, Lapika Dimonfu, Pinda Mukumbi, Mbongo Mpasi, Rev. Père Fridolin Aubongo, Kande Buloba, Munday Mulopo) approved the study retroactively. Animal studies were authorized by the agreement reference 75–759 from the French Ministry of Agriculture.

### 2. Development and optimization of the test

The dipstick was developed essentially as previously described [26]. To produce mAbs against the somatic antigen of *S. dysenteriae 1* [30], [31], BALB/c mice were immunized intraperitonally (i.p.) with 10^7^ CFU killed *S. dysenteriae* 1 bacteria three times at 3-week intervals. Mice eliciting the highest anti-LPS antibody response were given an intravenous boost injection 3 days before being sacrificed for splenic B cell fusion, according to Kohler and Milstein [30].

Hybridoma culture supernatants were screened for antibody (Ab) production by ELISA using LPS purified from *S. dysenteriae* 1, as previously described [26], [32], [33]. Briefly, LPS purified according to Westphal and Jann [34] was used at a concentration of 5 mg/ml in PBS. As secondary Abs, anti-mouse IgG-, IgM peroxidase-labeled conjugate (Sigma-Aldrich) were used at a dilution of 1/5,000. Only the hybridoma cells secreting IgG reacting specifically with LPS of the immunization strain, i.e., recognizing serotype-specific determinants on the LPS O-Ag, were selected. The selected hybridomas, representative of the four murine IgG subclasses, were then cloned by limiting dilution, and injected i.p. into histocompatible mice for ascitic production. IgG were precipitated with 50% ammonium sulfate from ascitic fluid, centrifuged, and dialyzed against PBS before being purified using ion-exchange chromatography as previously described [32], [33]. Among the available mAbs specific for *S. dysenteriae* 1, two IgG 2a were selected for the development of the RDSd1 test.

**Figure 1:**
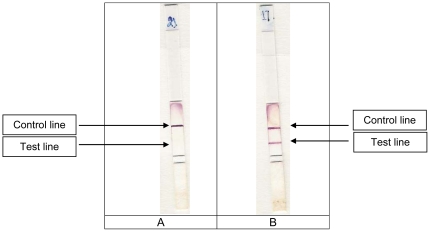
Two dispsticks showing typical negative (A) and positive (B) results after being kept for 10 minutes in bacterial cultures ^(1)^, watery or dysenteric stool samples. ^1^ The specificity was assessed using pure cultures of the following bacterial strains: *S. flexneri* serotypes 1a (strain 082429), 1b (strain 085052), 2a (strain 083766), 2b (strain 082831), 3a (strain 084963), 3b (strain 083638), 4 (strain 075519), 4c (strain 08 3649), 6 var Herforshire (strain 083400), 6 var Manchester (strain 080654), Y (strain 075876) and X (strain 08 3347); *S. dysenteriae* serotypes 2 (strain 083092), 3 (strain 081718), 4 (strain 083171), 5 (strain 071059), 6 (strain 087336), 11 (strain 9410434), 12 (strain 080360), 13 (strain 056376) and untypable strain 97–10607, a panel of six wild *S. dysenteriae* 1 strains from Central Africa [38] and five *S. dysenteriae* 1 wild strains from Centre National de Reference des Shigelles at Paris (strains 057331, 100771, 97171, 061306, 061305); *S. boydii* serotypes 1 (strain 07 7695), 2 (strain 08 3129), 3 (strain 07 8186), 4 (strain 08 3330), 5 (strain 599379), 6 (strain 346756), 8 (strain 06 6360), 9 (strain 0541), 10 (strain 081707), 11 (strain 065905), 12 (strain 06 8162), 13 (strain 161055), 14 (strain 08 0226), 15 (strain 04 8291), 17 (strain E3615 53), 18 (strain 078115), 19 (strain 07 5636), 20 (strain 08 2360); *S. sonnei* strain 08 7832 (phase 1) and strain 08 7785 (phase 2); *Salmonella enterica typhimurium* (strains 06-2835, 06-2846, 06-2847), *S enteritidis* (strains 06-2841, 06-2844, 06-2851, 06-2852), *S. hadar* (strains 06-2533), *S. brandenburg* (strain 06-2619), *S. heidelberg* (06-2843), *S. oranienburg* (strain 06-2634), *S. risen* (strain 06-2615), *S. stanleyville* (strain 06-2832), *S. typhi* (strain 06-2829), *S. paratyphi* A (strain 06-2633), *S. paratyphi* B (strain 06-2696), *S. meleagridis* (strain 06-2850), *S. stubra* (strain 06-2384), *S. huittingfoss* (strain 06-2391), enteroagregative *Escherichia coli* (strains 55989, JM221, O42, 56390 and 384P), diffusely adherent *E. coli* (strain AL851, AL847, C1845, AL855 and 3043), enterotoxigenic *E. coli* (strains EDL1496, 440TL, Tx-1, E2539-C1, 469), enteropathogenic *E. coli* (strains 135/12 (O55:H-), E6468/62 (O86:H34), 11201 (O125:H6), KK111/1 and F88/6848-2 both O26:H11), *E. coli* O148 (ref CNR E519-66), *Vibrio cholerae* O1 (strains CNRVC960255, 970002, 970014, 970025, 970067, 960325, 970022, 970053, 970055, 970056), *V cholerae* O139 (strains CNRVC 930008, 930381, 930210, 930190), *V. cholerae* non O1 and non O139 (strains CNRVC 930177, 930429, 950689, 950691, 970037, 950769, 910388, 930121, 930297, 930391), *V. alginolyticus* (strain CIP103336), *V. fluvialis* (strains CIP103355, CNRVC356), *V. parahaemolyticus* (strains CIP75.2, CNRVC-030478, CNRVC030479, CNRVC000204, CNRVC000208), *V. furnissii* (strain CIP102972), *V. hollisae* (strain CIP104354), *V. mimicus* (strain 101888), *Aeromonas caviae* (strain CIP76.16), *A. enteropelogenes* (strain CIP104434), *A. hydrophila* (strain CIP76.15), *A. sobria* (strain CIP74.33), *Plesiomonas shigelloides* (strain CIP63.5), *Campylobacter jejuni*, *Yersinia enterocolitica* 1A (6 strains of biotype 1A, 2 strains of biotype 2, 2 strains of biotype 3, and 2 strains of biotype I). Three rough wild *S. dysenteriae* 1 (strains 01587, 061305, 061306) were also tested.

The Rapid Diagnostic *S. dysenteriae* 1 (RDSd1) test is based on a one-step, vertical-flow immunochromatography using mAb-coupled colloidal gold particles [35]. The colloidal gold particles (40 nm diameter) were conjugated to the F24–70 anti-*S. dysenteriae* 1 mAb (British Biocell International Cardiff, UK) and lyophilised (A540 nm = 2) onto polyester release pads (Accuflow P Schleicher&Shull, Mantes la Ville, France). An automatic thin layer chromatography sampler (CAMAG 5, Muttenz, Switzerland) was used to spray the second selected anti-*S. dysenteriae* 1 mAb (B15–14) at a concentration of 2 µg/cm, as a line on nitrocellulose membrane (Immunopore FP, Whatmann International). In addition, a control capture line was obtained by spraying affinity-purified goat anti-mouse IgG (ICN Biomedical, Aurora, Ohio, USA), on a line higher up on the strip, at a concentration of 1 µg/cm. Cellulose filter paper was used for the wicking and sample pads (Cellulose paper 903, Schleicher&Shull). The immunostrips were then trimmed to a width of 5 mm (Figure 1) and stored in a waterproof bag (50 per desiccant bag) at 4°C before to be tested on stool samples and bacterial cultures.

With bacterial cultures in broth, the test was carried out in 5 ml disposable glass tubes at room temperature with a sample volume of 400 αl. After 10 minutes, a positive result appears as two strong red lines (upper control line and lower *S. dysenteriae* 1 LPS positive line), and a negative result as a single upper red control line (Figure 1). *S. dysenteriae* strain 1 (ref. NCDC-1007-71) was used as a positive control.

With stools, when liquid, a volume of about 400 µl (preferably including blood, mucus, rectal sputum when present) was transferred with a disposable pipette to a haemolysis glass tube of 5 ml. With semisolid stools, according to the consistency of the sample, a volume of about 200 to 400 µl of specimen was transferred into the haemolysis tube containing 100 to 300 µl of distilled water. With solid stools the RDSd1 test was performed on about 1 g of stools diluted in 400 µl of distilled water. The sample was always homogenised by several pipettings before the immunostrip was introduced in the test tube. RDSd1 tests must only be read when they are humid (never after drying) at an optimal time that is defined for each new batch of dipsticks. This optimal time was determined at the bench on reconstituted *S. dysenteriae* 1 positive stools and during a preliminary study on *S. dysenteriae* 1 positive and *S. dysenteriae* 1 negative stools.

### 3. Methodology of the RDSd1 test evaluation

The evaluations at the bench on strains and reconstituted stools and the evaluation on clinical stool samples were performed according to the STARD (Standards for Reporting of Diagnostic Accuracy) for new assays [36].

#### 3.1. Cut-off, reproducibility, shelf-life and specificity

Cut-off, reproducibility, shelf-life and specificity of the dipsticks on bacterial cultures were assessed by trained technicians. The cut-off (detection limit) and the range of detectable LPS concentrations was measured using two-fold dilutions (1 000 to 7.5 ng/ml of purified LPS) and tenfold dilutions of a *S. dysenteriae* 1 suspensions (5×10^3^ to 5×10^8^ bacteria/ml) using saline, and reconstituted stools (10 g of normal stool without *Shigella* spp suspended in 10 ml of saline). The reproducibility of the cut-off was assessed by testing ten strips of the same batch of RDSd1 tests simultaneously on a calibrated suspension of the *S. dysenteriae* 1 strain ref 100771. To predict the shelf-life of the RDSd1 test, we used the accelerated stability method that consisted in storing the assays for a given time at an elevated temperature [35]. The shelf-life of the strips in the laboratory was assessed by testing a dipstick three times per week for 10 weeks after storage at 25°C (air-conditioned room) or at 60°C (incubator). The specificity was assessed using pure cultures of the bacteria listed in Figure 1.

#### 3.2. Evaluation on clinical samples

The evaluation of the sensitivity and specificity of the RDSd1 test was performed in five clinical studies: in India at Chandigarh, Panjab and Haryana (Post Graduate Institute of Medical Education and Research (PGIMER)), in Senegal at Dakar (Institut Pasteur), in Vietnam at Ho Chi Minh City (Paediatric Hospital 1), in DRC at Kinshasa (Institut National de Recherche Biomédicale), in France at the Institut Pasteur (Investigation Clinique et Accès aux Ressources Biologiques) on healthy volunteers and in the field at Komanda in the DRC. The RDSd1 tests were shipped by air mail from France to India, Senegal, Vietnam and the DRC at ambient temperature in grip seal bags.

Stool samples were cultured for *Shigella* spp and other enteric bacterial pathogens and analyzed for parasites and viruses by using classical methods with minor modifications according to the laboratories [38]. Suspected colonies resembling *Shigella* were identified biochemically and serotyped by slide agglutination with monovalent O1 sera, according to the International *Enterobacteriaceae* Grouping Subcommittee [39]. When possible, samples that were positive by the dipsticks but negative by culture were evaluated by PCR of the *ipaH* gene of *Shigella* spp [40] and the Shiga toxin [41] afterward.

In Chandigarh, which is an area of dysentery endemicity involving *S. dysenteriae* 1, RDSd1 tests were evaluated during two periods (April to December 2007 and June to September 2009) of high incidence of the disease [37]. In 2007, 92 stools samples were collected during an outbreak of diarrhea and 40 from sporadic cases of dysentery, all from patients living in slums around Chandigarh. A total of 10 stool samples were collected from sporadic cases of dysentery in patients admitted to local dispensaries and in district hospitals in Chandigarh and the nearby states of Panjab and Haryana. In 2009, 43 stools were collected from patients consulting PGIMER for diarrheal disease. All these 185 stool samples were collected in sterile screw capped containers and immediately transported to the Medical Laboratory in PGIMER for diagnosis by classical methods by a trained technician. In 2007, aliquots of each of the 142 stools collected were frozen. A total of 86 frozen stool samples in which the aetiology was known were made available for this evaluation study from the specimen bank of the PGIMER. Stools were encoded. The RDSd1 tests were performed on defrosted stools by another trained technician. Results obtained with stool cultures for enteric pathogens and RDSd1 tests were then compared. In 2009, the second study was performed on 43 diarrheal fresh stools; stool cultures and the RDSd1 test were performed by two different technicians and the results were then compared.

In Dakar, the study was performed from June to August 2008. We compared the results obtained with stool cultures for enteropathogenic bacteria, viruses and parasites, and dipsticks performed in a blind study by two different technicians. A total of 75 stool samples from infants referred to the microbiology laboratory of Institut Pasteur of Dakar were collected. The dipstick test was performed by one technician and the stool samples were then tested for diagnosis by classical methods by another technician. Results obtained with stool cultures and dipsticks were then compared.

In France, 24 non-diarrheic stools from healthy volunteers consulting the Plate-Forme Investigation Clinique et Accès aux Ressources Biologiques (Institut Pasteur, Paris) were tested from February to April 2010. RDTSd1 were read by two persons and the results were compared at the end of the study.

In Ho Chi Minh City, a total of 53 stool samples from infants who had severe diarrhoea were collected and tested in the Paediatric Hospital I from November 2009 to March 2010. In a blind study, the results obtained with stool cultures and the RDSd1 tests performed by two different technicians were compared. Samples were then stored at -20°C.

In the DRC, during an outbreak of bloody diarrhoea at Zunguluka, district of Boga (Ituri, East Province) that occurred in July 2009, lactose-negative bacteria were isolated from 12 diarrhoeal stools in the laboratory of Ituri in the city of Bunia (located about 1 000 kms –East- of Kinshasa, 01°34′16.0 North/030°14′10.0 East) and sent to INRB in Kinshasa to be identified. Among these 12 stools, five were tested by using RDSd1 test in the laboratory. Strains sent to INRB were tested by using both RDSd1 tests and classical bacteriological methods for *Enterobacteriaceae*. In August 2009, during an outbreak of watery diarrhea at Ofaye, district of Komanda (Ituri, East Province) (01°13′10.4″ North/029°41′35″ East), RDSd1 tests were sent from INRB to the laboratory of Ituri in the city of Bunia (Laboratoire Médical de Référence de l′Ituri à Bunia) to be used in the field. A total of 43 stools were collected from adults and infants by using sterile screw-capped containers, transferred to 5 ml test tubes by using plastic pipettes (Pasteurette) and then subjected to the RDSd1 test. The collected stools were also preserved in Cary Blair and sent to the laboratory of Ituri in the city of Bunia. Lactose-negative colonies were isolated, preserved in Cary Blair medium and sent to Kinshasa to be identified at INRB. Results obtained with stool cultures and dipsticks were then compared.

#### 3.3. Statistics

We calculated the sensitivity (Se), which is the proportion of specimens with the target disorder in which the test result is positive; and the specificity (Sp), which is the proportion of specimens without the target disorder in which the test result is negative. The 95% confidence intervals (CI) for Se and Sp were determined [42]. We also calculated the Cohen's kappa (α) statistic [43] to measure concordance between stool culture and the RDSd1 test in the five prospective clinical studies. α may range from 0 to 1, and a α value of or higher than 0.8 is considered to indicate almost perfect agreement [44]. We also calculated likelihood ratios (LR). The positive LR (LR+  =  Se/[1 - Sp]) indicates how many times a positive result is more likely to be observed in specimens with the target disorder than in those without the target disorder. The negative LR (LR-  =  [1 - Se]/Sp) indicates how many times a negative result is more likely to be observed in specimens with the target disorder than in those without the target disorder. Accuracy increases the more the LR differs from 1. LR+ above 10 and LR- below 0.1 were considered convincing diagnostic evidence [45]. The diagnostic odds ratio (DOR), defined as the ratio of the odds of positive test results in specimens with the target disorder relative to the odds of positive test results in specimens without the target disorder, was calculated as follows [46]: DOR  =  (Se/[1 - Se])/([1 - Sp]/Sp). The DOR does not depend on prevalence and its value ranges from 0 to infinity, with higher values indicating better discriminatory test performance. The positive predictive value (PPV) represents the proportion of test-positive specimens that truly present the target disorder, while the negative predictive value (NPV) represents the proportion of test-negative specimens that truly do not present the target disorder: PPV  =  (P × Se)/(P × Se)+[(1 - P) × (1 – Sp)] and NPV  =  (1 - P) × Sp/[(1 - P) × Sp]+[P × (1 - Se)]. P is the prevalence of the target disorder in the population of specimens to which the test is applied. The 95% CI for PPV and NPV were also determined [45].

## Results

### 1. Cut-off, reproducibility and specificity on bacterial strains

The lower detection threshold of the dipstick for *S. dysenteriae* 1 LPS was 15 ng/ml in both distilled water and in reconstituted stools. Similar results were obtained using dipsticks stored for 22 days at 56°C. No prozone effect (i. e. no signal detected for high concentrations) was observed by using a range of LPS concentrations extending from 10 ng/ml to 1 mg/ml. In addition, in distilled water and in reconstituted stools containing different concentrations of *S. dysenteriae* 1, an unequivocal positive reaction was obtained in 2 minutes with 1.6×10^6^ CFU/ml and 4.9×10^6^ CFU/ml of *S. dysenteriae* 1, respectively (Figure 1). These detection limits were reproduced ten times. The specificity of the dipstick was 100% for all bacterial cultures with smooth strains listed in Figure 1. RDSd1 tests were negative with the three rough *S. dysenteriae* 1 strains.

### 2. Optimal time to read the test

**Figure 2:**
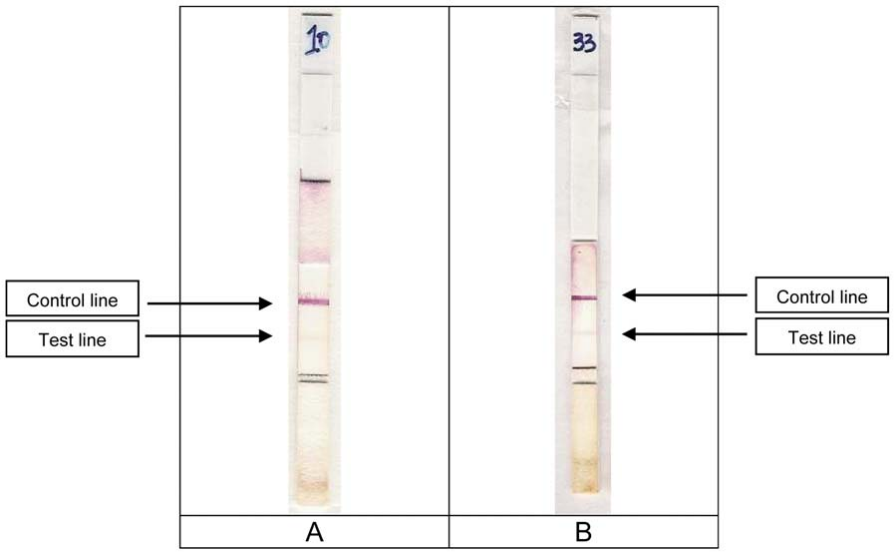
Two dipsticks showing a negative reaction with a faint yellow band appeared before or after the optimal time of 10 minutes to read the test (A) and after drying (B).

For bacterial cultures, the lower test line appeared in 1 to 2 minutes and the upper control line appeared 5–6 minutes later. On stools, for positive samples, the strong red lower positive line appeared in 2 to 3 minutes and a similar colour appeared on the upper control line 6 to 7 minutes later (Figure 1). With semisolid samples and stools containing mucus with a lower fluidity than bacterial cultures, the time required for soaking the dipstick was longer; consequently, the optimal delay to read the RDSd1 test was fixed at 10 minutes. During a preliminary study performed in India on 75 *S. dysenteriae* 1 negative liquid stools, a faint yellow or purple band was observed on the lower test line before the tenth minute on 25 (33.3 %) RDSd1 tests, and on 2 (2.6 %) after drying (Figure 2). For these reasons it has been stated that RDSd1 tests must never be interpreted over the defined optimal time for test and control lines, and never after drying. A sample is reported as positive if there is pink to red colour on the test line and on the control line in the optimal time.

### 3. Comparative prospective clinical studies

In the first study performed in Chandigarh, of the 86 patients from whom the stools were taken for the evaluation study the clinical characteristics of 85 patients are shown in Table 1. Clinical characteristics were not available for one RDSd1 test-and culture-positive patient. Of these 86 stool samples from patients in Chandigarh, 74 were both RDSd1 test-and culture-negative, 11 were RDSd1 test-positive before the tenth minute and culture-positive, and 1 was RDSd1 test-negative in the defined optimal time and stool culture positive. This last sample was clearly read dipstick-positive at the thirteenth minute. In the second study in 2009, all the 43 stools were both RDSd1 test and culture-negative. Among the 117 RDSd1 tests used on *S. dysenteriae* 1 negative stools, a faint yellow test band was observed before the tenth minute on 25 (21.4 %) RDSd1 tests and after the tenth minute on 5 (4.3 %) RDSd1 tests. A faint pink band on the test line was observed in one stool culture-negative sample after the dipstick was dried out of the test tube. These RDSd1 tests were interpreted as negative by the user.

**Table 1:** Clinical characteristics of patients from India (Chandigarh).

Characteristics	With *S. dysenteriae* 1	Others
	(n = 11)	(n = 74)
**Age (yr)**		
<5	2 (16.7)	23 (31.1)
6–14	2 (16.7)	17 (22.9)
15–45	6 (50)	23 (31.1)
45 and >	1 (8.3)	11 (14.9)
Mean+/− SD	24.5+/−21.6	19.7+/−20.02
Range	11 months to 75 yrs	5 days to 75 yrs
Watery Stool	8 (66.7)	62 (83.8)
Dysenteric stool (blood + mucus)	3 (25)	12 (16.2)
Vomiting	5 (41.7)	32 (43.2)
Fever	11 (91.7)	29 (39.2)
Abdominal pain	10 (83.3)	32 (43.2)
**Duration of diarrhea**		
<1	0 (0)	1 (1.35)
1–3	7 (58.3)	52 (70.2)
4–6	2 (16.7)	9 (12.2)
6–15	0 (0)	4 (5.4)
>15	2 (16.7)	8 (10.8)
**Dehydration status**		
None	7 (58.3)	51 (69)
Some	3 (25)	17 (22.9)
Severe	1 (8.3)	6 (8.1)
**Duration of stay at hospital or dispensary (h)**		
0–11	0 (0)	11 (15)
12–23	2 (16.7)	5 (6.7)
24–47	1 (8.3)	15 (20.3)
48–95	3 (25)	25 (33.8)
96+	5 (41.7)	18 (24.3)

Of the stool samples from patients in Dakar, 75 (100 %) were both RDSd1 test-and culture-negative. Faint yellow test bands appeared on 12 (16 %) dipsticks, and slight pink colour appeared on 8 (10.7 %) dipsticks after the optimal time; these 20 RDSd1 tests were interpreted as negative.

In Ho Chi Minh City, no *S. dysenteriae* 1 was isolated from the 53 studied stools. In one case the RDSd1 test failed (no colour on the control line) and was excluded from calculations. In 4 cases, the RDSd1 test was positive. A faint purple test band was observed on 3 negative *S. dysenteriae* 1 stools several minutes after the upper control line appeared and a very faint purple test band was observed in 8 (15 %) negative *S. dysenteriae* 1 stools in the prescribed time. These 11 RDSd1 tests were interpreted as negative.

In France, 24 stools were both RDSd1 test-and *S. dysenteriae* 1 culture-negative. A faint yellow test band was observed on 3 (12.5 %) dipsticks and on 3 other after drying. These 6 RDSd1 tests were interpreted as negative.

In DRC, during the first epidemic at Zunguluka, all the RDSd1 tests were negative on the stools and on the lactose-negative non *S. dysenteriae* 1 strains received and identified at INRB by classical methods. The 43 RDSd1 tests done in the field during the second epidemic were interpreted as negative by non-experienced health workers. No indeterminate results due to faint yellow colour (or another colour) on the test line was reported by the field technician.

**Figure 3:**
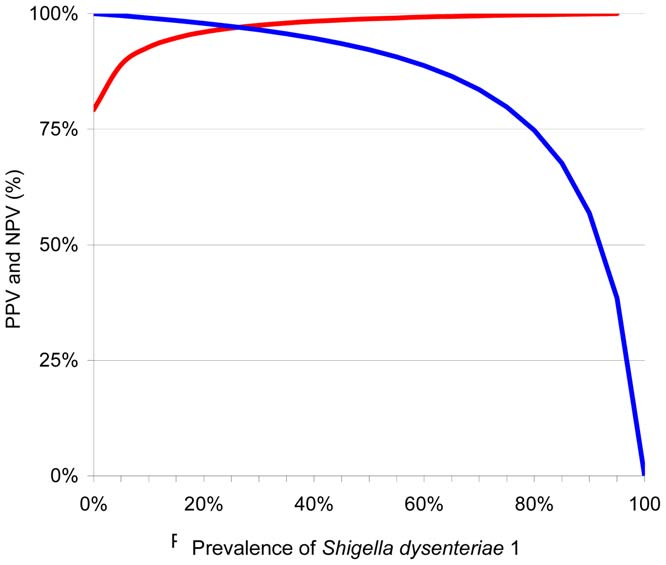
Predictive values (PV) for *Shigella dysenteriae* 1 diagnosis. Positive PV is represented by the red line and negative PV by the blue line.

Including all the data from the RDSd1 tests performed on stools (Table 2), specificity (312/316) was therefore 98.7% (95% CI: 96.6%–99.6%), the sensitivity (11/12) was 91.7% (95% CI: 59.8% – 99.6%). Stool cultures and RDSd1 tests gave concordant results in 98.4 % of cases (323/328) in the comparative studies. The Kappa coefficient obtained in this study was 0.8 ([0.984 – 0.921]/[1 – 0.921]). For the RDSd1 test, the LR+ was 70.5 and the LR- was 0.084, and the DOR was 839. The PPV (11/15) was 73.3% (95% CI: 44.8% – 91.1%) and the NPV (312/313) was 99.7% (95% CI: 98% – 100%). The variations of the PPV and the NPV according to prevalence were determined using the Se and Sp for clinical stool samples (Figure 3).

**Table 2:** Detection of *S. dysenteriae* 1 in 328 stool samples by RDSd1 test *versus* conventional culture.

Bacteriological culture	N° of specimens with *S. dysenteriae* 1 dipstick test result		
	Positive	Negative	Total
**Positive**	11 (India)	1 (India)	12
**Negative**	4 (Vietnam) 5	74 (India 2007) 1	316
		43 (India 2009) 2	
		75 (Senegal) 3	
		48 (DRC) 4	
		48 (Vietnam) 6	
		24 (France) 7	
**Total**	15	313	328

1 - 22 cultures were positive for *S. flexneri*, 20 for *Vibrio cholerae* O1, 5 for Enteroaggregative *E. coli*, 3 for *Salmonella enterica*, 2 for Enterotoxigenic *E. coli*, 2 for *Aeromonas* spp, 1 for Enteropathogenic *E. coli*, 1 for *Clostridium difficile*, 1 for *Pseudomonas aeruginosa*, 1 for *Cryptosporidium parvum*. In 52 RDSd1 test-negative samples *Shigella* spp was not isolated; on 38 of these samples *IpaH* PCR and Shiga toxin PCR were negative. For technical reasons (insufficient amount of stools, problems of storage), PCR was not done on 14 samples.

2 - A total of 14 cultures were positive for *V. cholerae* O1, 1 for *S. flexneri*, and 1 for Enteroaggregative *E. coli*.

3 - 30 stools were positive for parasites (8 *Endolimax nana*, 6 *E. coli*, 3 *Giardia lamblia*, 1 *E. histolytica*, 1 *Trichuris trichiura*, 1 *Hymenolepis nana*, 7 *E. nana and E. coli*, 1 *E. nana* and *Trichuris trichiura*, 1 *E. nana* and *E. histolytica*, 1 *E. nana* and *Ascaris lumbricoides*).

4 – During the first epidemic in Boga; 5 *Salmonella enterica* were identified among the lactose-negative strains received and studied at INRB. During the second epidemic at Ofaï, lactose-negative strains isolated at the dispensary of Bunia and sent to INRB were identified as *Salmonella enterica*.

5 - In one of these 4 samples, an Enteroinvasive *E. coli* and a classical Enteropathogenic *E. coli* were identified. No enteric pathogen was identified in the other 3 samples that were *IpaH* PCR negative.

6 - 1 culture was positive for *S. sonnei*, 6 were positive for diarrheogenic *E.coli* (4 Diffusely Adhering *E. coli*, 1 Enteroinvasive *E*. *coli*, 1 with both Diffusely Adhering *E. coli* and Enteroinvasive *E. coli*) and 2 for *Salmonella enterica*.

7 – No pathogen was identified.

## Discussion

*S. dysenteriae* 1 dysentery is often fatal without prompt and appropriate treatment [4]-[12]. Indeed, it causes a more severe and prolonged illness, particularly in young children, infants, the elderly, and the malnourished, than do infections with other *Shigella* serogroups [3], [5], [7]. Late diagnosis is one of the major causes of human death and spread of the disease since it limits the effectiveness of control measures. The review of the situation with regard to shigellosis led to a revision of the World Health Organisation guidelines for the control of bacillary dysentery [47]. The development of a reliable rapid diagnostic assay for improving diagnosis and surveillance is among the main modifications brought to these guidelines [22], [48]. The use of PCR assays based on the amplification of the invasion plasmid antigen H (*ipaH*) and Shiga toxin (*Stx*) gene sequences can overcome some of the shortcomings of culture methods but the method itself has not yet received global acceptance due to difficulties in its implementation in structures lacking microbiological support. The conventional culture methods currently used for diagnosis of *S. dysenteriae* 1 remains the gold standard but require a functioning laboratory and are time-consuming. Immunological methods for diagnosis of *Shigella* in stool samples have been studied [49], [50], [51], [52].

At this stage of the study, the RDSd1 test we developed and pre-evaluated has the following characteristics: quick time-to-answer, simple readout, able to be used by minimally trained personnel, the ability to function at 30° C and at high humidity, the ability to be stored for two years without refrigeration, the ability to conduct tests without the need for specific laboratory reagents (only water) or specialized laboratory equipment. The RDSd1 test was found to be highly specific when tested on bacterial cultures, with a better detection threshold (4.9×10^6^ CFU/ml of *S. dysenteriae* 1 and 15 ng/ml of LPS) than dipstick tests developed to diagnose cholera (10^7^ CFU/ml of *V. cholerae* O1 and 50 ng/ml of LPS) [18], [33], [25] and *S. flexneri* 2a infection (5×10^7^ CFU/ml, 20 ng/ml of LPS) [18], [33], [25]. Importantly, although the *E. coli* O148 O antigen repeating unit was shown to differ from that of *S. dysenteriae* type 1 only by the presence of a glucose residue in place of the galactose residue [30], no cross reaction was observed with *E. coli* O148:H28, which is one of the most common causes of diarrhoea in children in developing countries as well as in travellers to these areas [53].

This preliminary RDSd1 test evaluation on stools of patients living in endemic and non-endemic areas verified its high specificity. The RDSd1 test and stool cultures gave concordant results in 98.4 % and the kappa coefficient (0.8) obtained reflecting the good agreement. The NPV and the PPV exceeded 75 %, even during low prevalence of the disease. The public health implications of a very specific assay are greater in areas where the bacillary dysentery due to this serotype is rare than in countries were the disease is endemic, and a highly specific assay is most valuable to rule out *S. dysenteriae* 1 dysentery in an individual patient.

Given that antimicrobial therapy is recommended for all patients presenting symptoms of dysentery, the clinical significance of a positive rapid diagnostic *S. dysenteriae* 1 assay is high. Regarding the sensitivity of 91.7 %, the most likely explanation for the single discrepancy observed in India could be a very low concentration of *S. dysenteriae* 1 or LPS in the stool sample (because the test line was positive later, at the thirteenth minute). In Vietnam, among the four discordant results, it was not possible for one of them to conclude if there were co-infections because no PCR specific for *S. dysenteriae* 1 was available; in the three other samples, the presence of PCR inhibitors was suspected, but false-positive results with the RDSd1 test cannot be excluded. The dipstick test is sensitive enough when the lower test line is read at the third minute and when the upper control line is read seven minutes later. The sensitivity could be enhanced by reading after a longer time but we decided the optimal time is 10 minutes to avoid the potential risk of false positive results. In ten minutes, the great majority of the samples reached the wicking pad at the top of the dipstick. These two times depend on the distances between the sample pad and the test line, and between the sample pad and the control line. The shorter the distance, the shorter is the time of response.

A highly sensitive test is useful to alert medical authorities to an outbreak of *S. dysenteriae* 1 dysentery. At the beginning of an outbreak, critical interventions for *S. dysenteriae* 1 dysentery control include improved access to efficient treatment facilities, education to promote good personal hygiene, and improvement of sanitation and safe water supply. Successful interventions depend on early and easy detection of index cases. However, a suspected outbreak of *S. dysenteriae* 1 dysentery, whether detected by a rapid diagnostic assay or clinical diagnosis, should be confirmed with a stool culture from a sample of a typical patient. Culture confirmation will allow characterizing *S. dysenteriae* 1 strains and antimicrobial susceptibility testing to be performed on isolates in order to guide treatment. Furthermore, without assistance from a laboratory or a specific test, healthcare providers who are less accustomed to seeing patients with bacillary dysentery may have difficulties with differentiating bacillary dysentery from amoebiasis that presents quite similar symptoms but requires antiprotozoal therapy.

Although data from this preliminary evaluation on stool samples from patients living in endemic and non-endemic areas are encouraging, we need more positive stool specimens to definitively conclude on the sensitivity of the RDSd1 test. To estimate sensitivity of the dipstick with its 95% CI, a total of 100 *S. dysenteriae* 1 culture positive samples are needed in the evaluation panel. This full evaluation of the RDSd1 test faces the problem of the actual, but probably temporary, low prevalence of the disease in the world. A large-scale study comparing stool culture and RDSd1 test is warranted in the future.

Clinical data were only available for 85 patients recruited at Chandigarh. Interestingly, in this area, severe and milder forms of dysentery were developed by patients and included in the study. Dysenteric patients infected by *S. dysenteriae* 1 have generally a severe form of shigellosis with a clinical spectrum ranging from watery diarrhoea at the early stage of the infection to diarrhoea with mucus and frank bloody diarrhoea with fever beginning generally the third day of the infection. Bloody diarrhoea is associated with the rupture of the intestinal epithelial barrier, followed by the invasion and destruction of the intestinal mucosa, resulting in the proliferation of the pathogens faster than that occurring in patients with a milder form of the disease [54]. Patients who have the most severe form of shigellosis also shed a higher number of microorganisms [54]. A direct relationship between bacterial load (i. e. LPS concentration in stools), detection by culture, and disease severity also exists [54]. Consequently, it is essential to develop an efficient RDSd1 test that displays a low detection threshold and detects the somatic antigen without prozone effect to avoid false-negative results in samples containing high concentrations of *S. dysenteriae* 1 LPS antigen. We report here such a tool.

The dipstick must be read when it is humid and never after drying. A yellow faint or purple band appeared in the prescribed time on the test line of some dipsticks (17 %) tested on *S. dysenteriae* 1 negative sample. This phenomenon has been also observed for rapid diagnostic tests developed for cholera [55]. In our experience in the DRC, by informing of this phenomenon on the notice instruction, negative results were correctly read by the field technician working in the dispensary of Bunia. Because of the clear difference between the faint yellow or purple colour and the strong red or pink positive colour of the positive test band and of the control band, there was no misinterpretation by the operators during the study; furthermore the time to read the test was strictly respected, which is essential. An optimal time must be determined with each new batch of RDSd1 test. Investigations are in progress to know the origin of these faint or purple yellow colours observed on some test lines. We hypothesize that they may be a product of heme catabolism or are linked to the food regime.

This diagnostic test has been used in Chandigarh, Dakar, Ho Chi Minh City, Kinshasa and Paris, all sites with microbiological diagnosis infrastructures. The experience in the DRC at Bunia showed that a field technician could perform the dipstick assay in the dispensary and in the field (in the tropical forest) at least as well as was done in the laboratory, without specific training and only by following the technical notice; however, we need to confirm through other studies that these findings can be extrapolated to all laboratories and to field sites under different working conditions. According to this particular healthcare worker in the DRC, the dipsticks are easy to use in the field and after the test are performed, all the material can be sterilized either by chlorine or by burning them to avoid further risk of contamination. Data can be stored by digital photography.

Considering the potential impact this rapid diagnostic test will have for the clinical management of the disease and for early detection of *S. dysenteriae* 1 dysentery outbreaks, our group wishes to quickly develop collaborations with institutions located in areas in the developing world where *S. dysenteriae* 1 bacillary dysentery is endemo epidemic to definitively validate this RDSd1 test. Such a rapid diagnostic test could also allow better understanding of the burden of disease caused by this organism, therefore improving the evaluation of interventions. This diagnostic test has been mainly evaluated on sites with a microbiological diagnosis infrastructure. Although we have an experience in the field in the DRC, these findings cannot necessarily be extrapolated to sites where most reported bacillary dysentery cases occur. As it was previously realized for the immunochromatographic dipstick test specific for *V. cholerae* O1 [56], we therefore need to evaluate the RDSd1 test in an endemic setting typical of many urban or rural areas of developing countries and use conventional bacteriological culture as the reference standard.
